# Genomic basis of selective breeding from the closest wild relative of large-fruited tomato

**DOI:** 10.1093/hr/uhad142

**Published:** 2023-07-08

**Authors:** Junwei Yang, Yun Liu, Bin Liang, Qinqin Yang, Xuecheng Li, Jiacai Chen, Hongwei Li, Yaqing Lyu, Tao Lin

**Affiliations:** State Key Laborary of Agrobiotechnology, Beijing Key Laboratory of Growth and Developmental Regulation for Protected Vegetable Crops, College of Horticulture, China Agricultural University, Beijing 100193, China; State Key Laborary of Agrobiotechnology, Beijing Key Laboratory of Growth and Developmental Regulation for Protected Vegetable Crops, College of Horticulture, China Agricultural University, Beijing 100193, China; State Key Laborary of Agrobiotechnology, Beijing Key Laboratory of Growth and Developmental Regulation for Protected Vegetable Crops, College of Horticulture, China Agricultural University, Beijing 100193, China; State Key Laborary of Agrobiotechnology, Beijing Key Laboratory of Growth and Developmental Regulation for Protected Vegetable Crops, College of Horticulture, China Agricultural University, Beijing 100193, China; State Key Laborary of Agrobiotechnology, Beijing Key Laboratory of Growth and Developmental Regulation for Protected Vegetable Crops, College of Horticulture, China Agricultural University, Beijing 100193, China; State Key Laborary of Agrobiotechnology, Beijing Key Laboratory of Growth and Developmental Regulation for Protected Vegetable Crops, College of Horticulture, China Agricultural University, Beijing 100193, China; State Key Laborary of Agrobiotechnology, Beijing Key Laboratory of Growth and Developmental Regulation for Protected Vegetable Crops, College of Horticulture, China Agricultural University, Beijing 100193, China; Shenzhen Branch, Guangdong Laboratory of Lingnan Modern Agriculture, Genome Analysis Laboratory of the Ministry of Agriculture and Rural Affairs, Agricultural Genomics Institute at Shenzhen, Chinese Academy of Agricultural Sciences, Shenzhen, 518124, China; State Key Laborary of Agrobiotechnology, Beijing Key Laboratory of Growth and Developmental Regulation for Protected Vegetable Crops, College of Horticulture, China Agricultural University, Beijing 100193, China

## Abstract

The long and intricate domestication history of the tomato (*Solanum lycopersicum*) includes selection sweeps that have not been fully explored, and these sweeps show significant evolutionary trajectories of domestication traits. Using three distinct selection strategies, we represented comprehensive selected sweeps from 53 *Solanum pimpinellifolium* (PIM) and 166 *S. lycopersicum* (BIG) accessions, which are defined as pseudo-domestication in this study. We identified 390 potential selection sweeps, some of which had a significant impact on fruit-related traits and were crucial to the pseudo-domestication process. During tomato pseudo-domestication, we discovered a minor–effect allele of the *SlLEA* gene related to fruit weight (FW), as well as the major haplotypes of *fw2.2*/*cell number regulator* (*CNR*), *fw3.2*/*SlKLUH*, and *fw11.3*/*cell size regulator* (*CSR*) in cultivars. Furthermore, 18 loci were found to be significantly associated with FW and six fruit-related agronomic traits in genome-wide association studies. By examining population differentiation, we identified the causative variation underlying the divergence of fruit flavonoids across the large-fruited tomatoes and validated *BRI1-EMS-SUPPRESSOR 1.2* (*SlBES1.2*), a gene that may affect flavonoid content by modulating the *MYB12* expression profile. Our results provide new research routes for the genetic basis of fruit traits and excellent genomic resources for tomato genomics-assisted breeding.

## Introduction

Artificial selection of vegetable crops has changed human dietary habits during the last 10,000 years. The impacts of the long and intricate history of pseudo-domestication on the tomato (*Solanum lycopersicum*) could be manifested in the tomato genome sequence [1]. The history of tomato pseudo-domestication has been described as a “two-step” process, beginning with domestication from the blueberry-sized *S. lycopersicum* var. *pimpinellifolium* (PIM) to the cherry-sized *S. lycopersicum* var. *cerasiforme* (CER), and improvement from CER to the large-fruited *S. lycopersicum* var. *lycopersicum* (BIG) [2, 3]. However, CER, a weedy or wild species from central South America, may have existed before tomato domestication because of its complicated genetic mixing of both PIM and BIG [4, 5].

Larger fruits, improved taste, and an overall more robust plant were selected throughout tomato evolution because they are valuable to humans and essential for plant survival [6]. One of the most crucial agronomic traits, fruit weight (FW), has been selected by human for hundreds of years. Several genes/quantitative trait loci (QTLs) controlling fruit size were selected and identified during the consecutive domestication and breeding of tomato [7–11], including *fw2.2*/*cell number regulator* (*CNR*), *fw3.2*/*SlKLUH*, *fw11.3*/*cell size regulator* (*CSR*), *lc/WUSCHEL* (*SlWUS*), and *fas/CLAVATA3* (*SlCLV3*). By changing the cell division rate, cell number, and meristem size during fruit development, these genes increase the cell layer and carpel/locule number (LN). The first cloned fruit mass gene, *fw2.2*/*CNR*, negatively regulates cell division and accounts for 30% of fruit size variation contributing to increased FW [7]. The second fruit mass gene, *fw3.2*/*SlKLUH*, primarily affects the pericarp and septum in large-fruited tomatoes by increasing the cell number [8]. The third fruit mass QTL, *fw11.3*/*CSR*, explains about 8% of the phenotypic variation [9]. Briefly, cell division and expansion are main drivers of organ growth in plants. In addition, *SlWUS* and *SlCLV3* participate in the WUS–CLV pathway, which controls fruit size in tomato by regulating the LN [11, 12].

In addition, selection of many other important morphological traits, including some specific metabolites [13], inflorescence architecture, LN, and fruit shape, has been accompanied by dramatic, relatively rapid changes in fruit size. The artificially selected *fw11.3* gene hitchhiked during tomato domestication, altering the content of eight metabolites and thus affecting fruit quality [13]. Several genes play a crucial role in increasing tomato yield, among which *SINGLE FLOWER TRUSS* (*SFT*) is the antagonist of *SELF PRUNING* (*SP*), which could increase tomato yield [14]. In order to increase fruit size, the AP2/ERF transcription factor *EXCESSIVE NUMBER OF FLORAL ORGANS* (*ENO*) shows synergistic effects with *SlWUS* and *SlCLV3* [15]. Furthermore, it was discovered that the *GLOBE* gene, which encodes brassinosteroid hydroxylase, determines the shape and size of the tomato fruit [16].

The highly diversified crop metabolome is often regarded as a link between the genome and the phenome. Breeders use a new strategy called the integrative analysis of multi-omics data to investigate the genetic basis of crop metabolic and agronomic traits. Several loci for tomato fruit quality traits have been identified, including sucrose, ascorbic acid, malic acid, citric acid, and volatiles [17–19]. Metabolomics data were used to predict the performance of several agronomic traits in wheat (*Triticum aestivum*), including the grain number per spike and plant height [20]. In addition, 36 candidate genes were found to regulate the levels of metabolites that are of potential physiological and nutritional importance in rice (*Oryza sativa*) [21]. Meanwhile, 1035 metabolomes were utilized to analyze the natural variation and genetic control of maize drought adaptation in maize (*Zea mays*) [22].

In this study, we performed EigenGWAS, nucleotide diversity analysis, and the cross-population composite likelihood ratio test (XP-CLR) strategies on the PIM and BIG groups to identify selective sweeps during tomato pseudo-domestication. In total, we identified 390 putative selective sweeps, some of which were critical to the pseudo-domestication process and affected fruit-related traits. Also, we detected six plausible candidate loci that may have an impact on fruit mass during tomato pseudo-domestication. In addition, GWASs identified * BRI1-EMS-SUPPRESSOR 1.2 (SlBES1.2)*, a transcription factor of BES1 family that may affect flavonoid content by regulating *SlMYB12* expression, as well as the causative variation that may cause the divergence of fruit flavonoids among the large-fruited tomatoes. Our findings offer valuable data for illustrating the genetic architecture of tomato fruit mass and metabolite content, which will boost tomato breeding efforts in future.

## Results

### Pseudo-domestication from the PIM to BIG group

**Figure 1:**
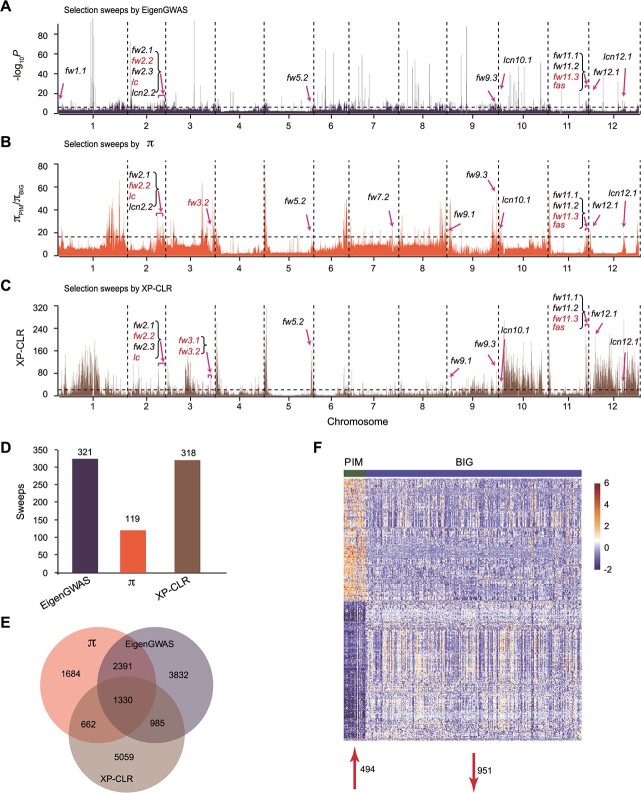
**Genome-wide scanning in the PIM and BIG groups using three distinct strategies. (A–C)** A total of 321 (top 5%, *P* ≤ 4.00 × 10^−07^), 119 (top 5%, π_PIM_/π_BIG_ ≥ 16.46), and 318 (top 5%, XP-CLR ≥ 21.56) regions were considered candidate pseudo-domestication sweeps using EigenGWAS(A), π **(B)**, or XP-CLR **(C)**, respectively. **(D–E) **The number of selective sweeps** (D)** and the number of genes within these selective sweeps** (E)** detected by the three different strategies.** (F)** Heat map of the genes expressed differentially within selected regions in the PIM and BIG groups based on normalize FPKM values using log_2_(FPKM + 1).

In this study, we explored the phylogenetic relationships among 219 diverse accessions, including 53 wild tomatoes (*Solanum pimpinellifolium*; PIM) and 166 large-fruited cultivars (*S. lycopersicum* var. *lycopersicum*; BIG) using 46,850 single nucleotide polymorphisms (SNPs) (minor allele frequency [MAF] >5%, missing data <10%, and *r*^2^ threshold <0.2). The neighbor-joining tree largely approves the division of PIM and BIG groups (Supplemental Fig. 1). To identify comprehensive selective sweeps of tomato in the process of pseudo-domestication, we performed three genomic analyses: EigenGWAS, nucleotide diversity (π), and the XP-CLR test on these accessions. We identified a total of 390 putative pseudo-domestication sweeps covering 329.32 Mb (43.34%) of the assembled genome in tomato, consisting of 321 (*P* ≤ 4.00 × 10^−7^) covering 106.35 Mb through EigenGWAS (PDS_E_), 119 (π_PIM_/π_BIG_ ≥ 16.46) covering 62.7 Mb through π (PDS_π_), and 318 (XP-CLR ≥ 21.56) covering 219.33 Mb through XP-CLR (PDS_X_), respectively (Fig. 1A–D and Supplemental Tables 1–4).

Using the three strategies, a total of 15,942 genes were identified in these selective sweeps (Supplemental Tables 5–7). Among these genes, 1,330 pseudo-domestication genes were identified by three strategies simultaneously. In addition, we found that 3,721 and 2,315 PDS_E_ genes overlapped with PDS_π_ and PDS_X_ genes, respectively, and 1,992 PDS_π_ genes overlapped with PDS_X_ genes (Fig. 1E). Furthermore, we found that 36 genes or loci were located in these potential pseudo-domestication sweeps, including several related to fruit mass and LN (Supplemental Table 8). Among these 36 loci, two major genes related to fruit mass, *fw2.2* and *fw11.3*, were consistently located within pseudo-domestication sweeps using these three strategies, whereas *fw3.2* was identified by only two of the three (π and XP-CLR) (Fig. 1A–C). Our results indicate that these three strategies can be combined for the study of tomato pseudo-domestication.

To assess the variation of the transcriptome level during tomato pseudo-domestication, we estimated gene expression distribution and transcript abundance for the PIM and BIG groups from a previous report [13]. A total of 4,724 differentially expressed genes (DEGs) were detected between the PIM and BIG groups (Supplemental Fig. 2), of which 1,445 were selected by humans during tomato pseudo-domestication, including 951 down-regulated and 494 up-regulated genes (Fig. 1F). GO analysis showed that these DEGs were involved (*P* < 0.01) in the following biological processes: oxidation–reduction, glutamate metabolic, glutamine family amino acid metabolic, molecular function regulator, and enzyme inhibitor/regulator activities (Supplemental Fig. 3A and Supplemental Table 9). Furthermore, Kyoto Encyclopedia of Genes and Genomes (KEGG) analysis revealed that these genes were enriched in pathways including riboflavin and tyrosine metabolism, phenylpropanoid biosynthesis, phagosome, and limonene and pinene degradation (Supplemental Fig. 3B and Supplemental Table 10). Furthermore, among these DEGs, we also discovered the *Solyc03g097580* and *Solyc03g097870* for glucose content [19], *Solyc04g015530* (*ps-2*) for functional sterility [23], *Solyc06g074240* (*B/OG*) for β-carotene [24], and *Solyc12g008980* (*Del*) for carotenoid biosynthesis [25], indicating the importance of these DEGs in tomato pseudo-domestication.

### Genomic selection for FW during tomato pseudo-domestication

**Figure 2:**
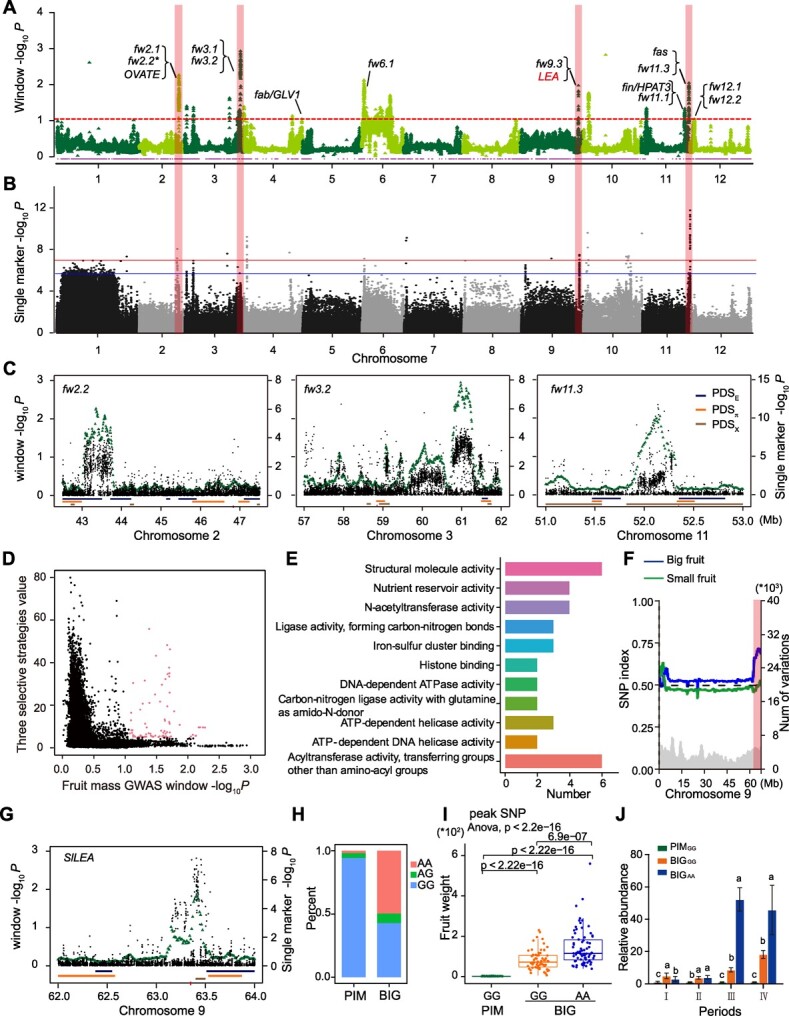
**Genome-wide association analysis for FW. (A)** GWAS for FW in the PIM and BIG groups. The triangles above the threshold line show windows in the top 1% from GWAS. **(B)** Single marker (−log_10_) *P* value for GWAS on FW (*λ* = 0.321). **(C) **Local Manhattan plot for FW-related genes *fw2.2*, *fw3.2* and *fw11.3*. **(D)** Three selective strategies value with fruit mass GWAS *P* values averaged over 100-kb windows. Pink color-highlighted points correspond with those highlighted regions in** (A)**. **(E)** GO analysis of FW-related genes detected by the three selective strategies. **(F)** Local SNP indices for chromosome 9. **(G)** Local Manhattan plot for FW-related candidate gene *Solyc09g082110* (*SlLEA*). **(H)** Allele frequency of the selected SNP (chr09:63405303) of *LEA* in PIM and BIG groups. **(I)** Statistical analysis of FW for the *LEA* genotypes among the PIM and BIG groups. **(J)** The relative expression profile of *SlLEA* from pre-anthesis (I), full-bloom stage (II), and 5 days post-anthesis (III) to 10 days post-anthesis (IV) in the PIM and BIG groups.

After spreading from the Andes Mountains in South America to the rest of the world, the fruit mass and quality of tomato fruit have improved significantly from PIM to BIG lines. Some key genes for these traits have been identified, including *fw2.2*, *fw3.2*, *fw11.3*, *fas*, *sun*, and *lc*. However, the genomic selective characteristics related to fruit mass during tomato pseudo-domestication have not been thoroughly explored. To identify potential selection signals, we analyzed pseudo-domestication sweeps related to fruit mass together with GWAS results (Fig. 2A–C). A total of 40 significant outlier regions were identified during tomato pseudo-domestication, accounting for 13.27 Mb of the tomato reference genome (Fig. 2A). Further analysis found that 976 candidate genes were located within these outlier regions, and 171 genes were located in swept regions identified by the three strategies (Fig. 2A and D). Through GO analysis of these candidate genes, their function showed in structural molecule, nutrient reservoir, and N-acetyltransferase activity (Fig. 2E). Intriguingly, three known genes, *fw2.2*, *fw3.2*, and *fw11.3*, were found around the peak SNPs on chromosomes 2, 3, and 11, respectively, and were located within pseudo-domestication sweeps (Fig. 2C).

To identify the regions related to fruit mass, we constructed an F_2_ segregating population using a cross between the PIM (TS-19) and the BIG (TS-400) tomato accession. Using bulked segregant analysis of the F_2_ population, we found one significant interval with FW at the distal end of chromosome 9 (Fig. 2F). In addition, we identified one significant outlier region (*P*_SNP:chr9:63405303_ = 3.14 × 10^−8^ and *P*_window:chr9:63370000_63,470 000_ = 1.973; around 63.32–63.51 Mb) on the pseudo-domestication sweep (PDS_E263_ = 8.29, PDS_X255_ = 38.80) of chromosome 9 (Fig. 2G). Through functional analysis, a candidate gene (*Solyc09g082110*) encoding a seed maturation protein/late embryogenesis abundant (SlLEA) protein was located within this interval (Fig. 2G). The haplotype analysis showed that *Solyc09g082110* had one major haplotype (GG) in the PIM group, whereas there were two haplotypes (AA and GG) in the BIG group (Fig. 2H). Furthermore, in the BIG group, we found that compared with haplotype GG, haplotype AA of *Solyc09g082110* significantly increased the FW (Fig. 2I). The experimental analysis showed that the expression levels of *Solyc09g082110* of the BIG accessions carrying haplotype AA were higher than those harboring haplotype GG during the fruit expansion stages (Fig. 2J and Supplemental Fig. 4A). Meanwhile, we found that haplotype AA could produce more locules (Supplemental Fig. 4B and C). These results suppose that *Solyc09g082110* could be a candidate gene for determining fruit mass by altering the LN in the BIG group, and the haplotype AA/GG might affect the transcriptional level of *Solyc09g082110* at the fruit expansion stage. However, the mechanism and causal variation of *Solyc09g082110* need to be further validated functionally.

### Selection of agronomic traits related to FW

**Figure 3:**
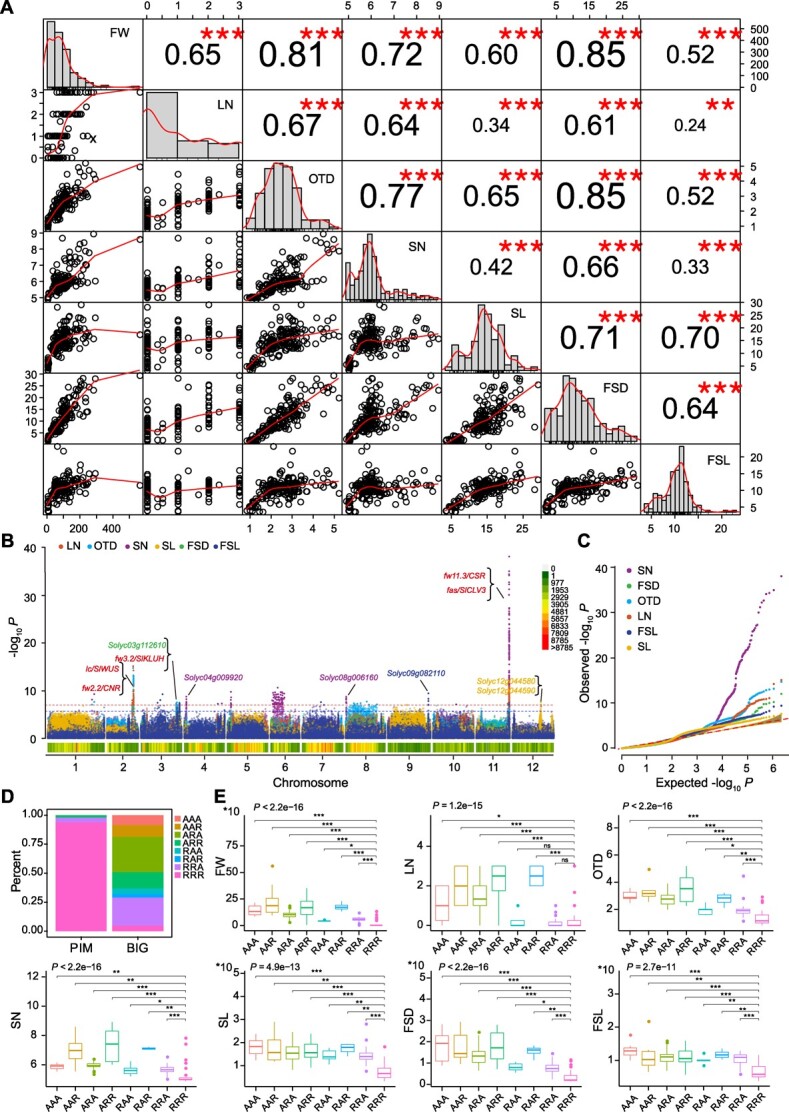
**Genome-wide associations for six agronomic traits related to FW. (A)** Correlation and Gaussian distribution of FW (FW), LN, OTD, SN, SL, FSD, and FSL.** (B–C)** Manhattan plot **(B) **and quantile–quantile (Q–Q) plot **(C)** of GWAS for LN (*λ* = 0.608), OTD (*λ* = 0.326), SN (*λ* = 0.319), SL (*λ* = 0.395), FSD (*λ* = 0.263), and FSL (*λ* = 0.661). **(D)** Allele distribution of FW-related SNPs at positions Chr02:41796211, Chr03:57938377, and Chr11:52136718 in the PIM and BIG groups. **(E) **Allele distribution in varieties containing R or A allele combinations associated with agronomic traits related to FW, LN, OTD, SN, SL, FSD, and FSL. R is the PIM allele and A is the alternate allele.

In the process of tomato pseudo-domestication, humans tend to select those larger and tastier fruits, which are accompanied by change of inflorescence architecture. To reveal the selection of agronomic traits related to FW, we exploited 28 agronomic traits for 219 diverse tomato accessions, divided into three categories: plant architecture, floral architecture, and fruit mass. We found that six traits, including LN, ovary transverse diameter (OTD), sepal number (SN), sepal length (SL), fruit stalk diameter (FSD), and fruit stalk length (FSL), were highly correlated (*r* > 0.5) with FW (Fig. 3A) and exhibited significant differences between the PIM and BIG groups (Supplemental Fig. 5).

In the PIM and BIG groups, we performed large-scale GWASs on these six agronomic traits highly related to FW (Fig. 3B and C). A total of 18 significant signals (*P* < 1.05 × 10^−7^) were identified in the tomato genome (Fig. 3B and Supplemental Table 11). Among these signals, five were shared among FW and other traits, including *fw2.2* for FSD, OTD, and LN on chromosome 2; *fw3.2* for FSD and OTD on chromosome 3; and *fw11.3* for SN, OTD, LN, and FSL on chromosome 11 (Fig. 3B). Furthermore, haplotype analysis on the three FW-related loci (Chr02:41796211, Chr03:57938377, and Chr11:52136718) showed eight major haplotypes in the BIG group (Fig. 3D). Among these haplotypes, the RRR haplotype is predominant in the PIM group, and we found that the major haplotype (AAA) in the BIG group contributed to the larger fruit and greater organ number (Fig. 3E). Interestingly, five novel loci related to SN, FSD, OTD, FSL, and SL were identified (Supplemental Table 11). The above results indicate that tomato FW and the agronomic traits related to FW may have shared a part of common genetic basis during the process of tomato pseudo-domestication.

### Differentiation of flavonoid content in large-fruited tomatoes

**Figure 4:**
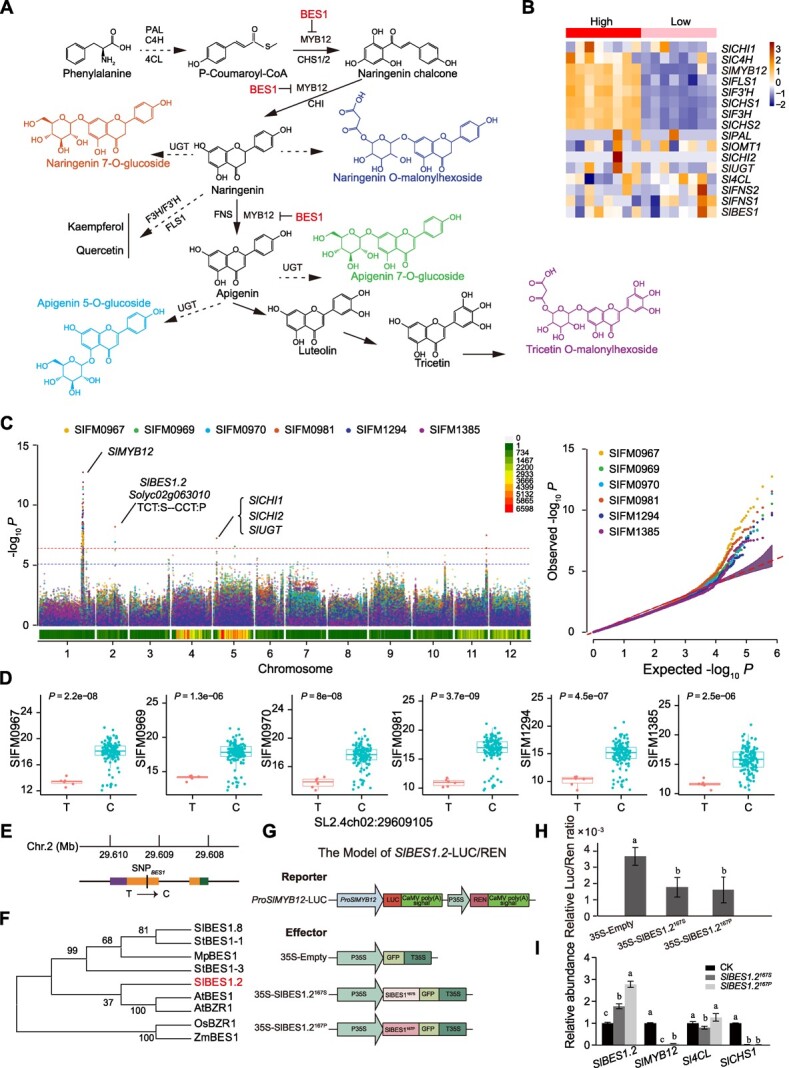
**Genome-wide associations for six flavonoid traits. (A)** Representative flavonoid biosynthetic pathway. This pathway takes naringenin chalcone as the precursor and is produced from phenylalanine. PAL: phenylalanine ammonia lyase, C4H: cinnamate-4-hydroxylase, 4CL: 4-coumarate-coenzyme A ligase, CHS: chalcone synthase, CHI: chalcone isomerase, MYB12: MYB DOMAIN PROTEIN 12, UGT: uridine diphosphate-dependent glucosyltransferase. **(B)** Heat map of genes involved in the representative flavonoid biosynthetic pathways in high/low flavonoid tomato accessions from the BIG group. **(C) **Manhattan plot and quantile–quantile (Q–Q) plot of GWAS for SIFM0967 (heptamethoxyflavone, *λ* = 0.810), SIFM0969 (apigenin 7-*O*-glucoside, *λ* = 0.806), SIFM0970 (apigenin 5-*O*-glucoside, *λ* = 0.792), SIFM0981 (naringenin 7-*O*-glucoside, *λ* = 0.757), SIFM1294 (naringenin *O*-malonyl hexoside, *λ* = 0.819), and SIFM1385 (tricetin *O*-malonyl hexoside, *λ* = 0.878). **(D)** Boxplot of six flavonoid traits related to FW (FW) in tomato accessions with different alleles (T or C) of *SlBES1.2* at SNP chr02:29609105. **(E) **Gene model of *SlBES1* and mutation (SNP) at position 167 bp. **(F)** Phylogenetic tree of SlBES1.2 and its homologues in tomato (*S. lycopersicum*), potato (*S. tuberosum*), *A. thaliana*, rice (*O. sativa*), maize (*Z. mays*), and *Marchantia polymorpha*. **(G)** Schematic diagram of the reporter and effector constructs used for the dual-luciferase (LUC) reporter assay. **(H)** Relative LUC/REN ratios were measured. Significant differences were determined using Student *t* test.** (I)** Relative expression of *SlBES1.2*, *SlMYB12*, *Sl4CL*, and *SlCHS1*. Bars show mean values and error bars represent SD (*n* = 3).

Flavonoids are an important metabolite that determinants tomato fruit quality, which can affect human consumption and acceptability. During tomato breeding, these compounds have gradually become the key indicators for farmers and breeders to cultivate tomato varieties. However, the genetic basis of divergence of flavonoid biosynthesis has not been fully studied among large-fruited tomatoes. In this study, we found that a series of genes involved in flavonoid biosynthesis, including *cinnamate-4-hydroxylase* (*SlC4H*), *4-coumarate-coenzyme A ligase* (*Sl4CL*), *MYB12* (*SlMYB12*), *chalcone synthase 1* (*SlCHS1*), *chalcone synthase 2* (*SlCHS2*), *flavanone 3-hydroxylase* (*SlF3H*), *flavanone 3′-hydroxylase* (*SlF3′H*), and *flavonol synthase 1* (*SlFLS1*), were more highly expressed in high flavonoid-accumulating tomatoes than those with low flavonoid-accumulating content (Fig. 4A and B).

In order to further understand the genetic basis underlying the divergence of flavonoid in the BIG group, we performed a single-trait GWAS on 166 large-fruited accessions for six flavonoid-related metabolites, including glycosides (SIFM0981, SIFM1294, SIFM0969, SIFM0970, and SIFM1385) and heptamethoxyflavone (SIFM0967) (Supplemental Table 12). A strong association signal was identified on chromosome 1, with the highest −log[*P*] value among these six metabolites, and resided upstream of *SlMYB12*. In addition, we detected *Solyc02g063010*, encoding the transcription factor BRI1-EMS-SUPPRESSOR 1.2 (SlBES1.2) [26], *Solyc05g010310* and *Solyc05g010320*, encoding Chalcone isomerase 1 and Chalcone isomerase 2 (SlCHI1 and SlCHI2), respectively [27, 28], as well as *Solyc05g012660*, encoding a UDP-glycosyltransferase (SlUGT) (Fig. 4C).

In the coding region of *Solyc02g063010/SlBES1.2*, we found that a polymorphism at position 167 bp (SNP*_BES1.2_* T/C) caused the substitution from serine to proline in SlBES1.2 (Fig. 4D and E). According to the haplotype analysis, the CC allele (proline) was found to mainly exist in high flavonoid-accumulating accessions (Fig. 4D). Interestingly, the *Arabidopsis thaliana* ortholog of *SlBES1.2*, *AtBES1*, could repress *MYB12* expression and reduce flavonoid biosynthesis in *Arabidopsis* [29]. The phylogenetic tree of homologous proteins showed that *SlBES1.2* had a similar function to *AtBES1* [29], indicating that *SlBES1.2* played a crucial role for the differentiation of flavonoids in tomato divergence (Fig. 4F).

To investigate whether *SlMYB12* is regulated by SlBES1.2, we conducted a dual-luciferase reporter assay, consisting of a reporter construct containing the *SlMYB12* promoter and effector constructs carrying either *SlBES1.2^167S^* or a mutated *SlBES1.2^167P^* (Fig. 4G). Co-transformation experiments in *Nicotiana benthamiana* leaves showed significantly lower LUC activity than the control (Fig. 4H). To verify the causative role of this gene in varying tomato flavonoid content, we used high/low flavonoid tomato lines overexpressing *SlBES1.2* (*SlBES1.2*-OE). We found that the relative expression of flavonoid-related genes, such as *SlMYB12* and *SlCHS1*, decreased in the *SlBES1.2*-OE lines (Fig. 4I). Taken together, our results indicate that the transcription factor *Solyc02g063010/SlBES1.2* is involved in the tomato flavonoid-biosynthesis-related metabolic pathways.

## Discussion

As the world’s most important vegetable, the commercial tomato contains more nutrients than its wild germplasm, including abundant soluble solids, flavonoids, vitamins, and antioxidants [30]. In order to improve the flavor of tomato fruit and increase its resistance to pathogens, genomic fragments from the wild germplasm were introgressed into cultivated tomato during breeding [2, 31]. The evolution of tomato genome is described as a two-step process with an increase in fruit mass: from PIM to CER, and then from the CER to BIG groups [2]. However, the CER in South America is native to the Ecuadorian and Peruvian Andes, and is considered as an evolutionary intermediate between the PIM and BIG groups [2], or alternatively, an admixture produced by extensive hybridization [4, 5, 32, 33]. To better understand the genetic mechanism of tomato domestication, we studied the small-fruited wild tomatoes (*S. pimpinellifolium*) native to the Andean regions of South America and the large-fruited cultivated tomatoes grown worldwide.

FW is one of the most important quantitative inherited traits controlled by multiple genetic loci. To date, researchers have found about 30 QTLs related to fruit size and shape [2, 34, 35]. However, only three genes affecting FW were found in tomatoes, including *fw2.2*/*CNR*, *fw3.2*/*SlKLUH*, and *fw11.3*/*CSR*[7–9]. In our study, we found a leading SNP (chr09:63405303) in the *fw9.3* locus on chromosome 9 by analyzing the PIM and BIG groups. A candidate gene (*Solyc09g082110*) encoding a seed maturation/late embryogenesis abundant (LEA) protein was found in this locus. Previous studies have shown that the *LEA* gene indeed regulated plant growth and organ development. Orthologs of *Solyc09g082110* play a pivotal role in osmotic regulation and salt stress response in wheat [36]. In addition, the rice LEA protein HVA1 promotes root development through an auxin-dependent process [37], and the *Brassica napus LEA3* could improve photosynthetic efficiency to increase the accumulation of oil content in seeds [38]. Furthermore, *TaHVA1* improved the biomass productivity and water use efficiency in wheat [39]. In this study, we also found that the mutation of the leading SNP upstream of the *SlLEA* gene was significantly associated with FW. During the expansion stage of the ovary, the relative expression level of this gene was upregulated significantly in the BIG_AA_ group compared to the BIG_GG_ group. We hypothesized that this leading SNP could regulate the expression level of the *Solyc09g082110* and further influence fruit enlargement during the developmental stage of the ovary in the BIG group.

After the pseudo-domestication of tomato, different tomato varieties were developed in accordance with human preferences. Among these diversified varieties, pink tomatoes are preferred, particularly popular with consumers in Asia. Meanwhile, hundreds of metabolites were modified during the pink tomato breeding [13]. Flavonoids accumulate in tomato fruits and contribute to its fruit color and ultraviolet protection [28]. In addition to the previously reported gene *SlMYB12* [40], we also identified a candidate gene (*Solyc02g063010*) encoding the transcription factor BRI1-EMS-SUPPRESSOR 1.2 (SlBES1.2), which is the primary regulator of brassinosteroid signaling transduction. In plants, BES1 could directly inhibit biosynthesis of the brassinosteroids and jasmonates by interacting with several MYB genes, thus influencing the growth–defense tradeoff [41]. In *Arabidopsis*, BES1 negatively regulated the expression level of the transcription factor, *MYB11*, *MYB12*, and *MYB111*, in flavonoid biosynthesis [29], which is consistent with the *MYB12* expression pattern of and flavonoid content in tomato. Moreover, the previous studies reported that BRASSINOSTEROID-INSENSITIVE2 (BIN2) kinase could phosphorylate BES1 and inhibit its activity [42], which indicates that the content of flavonoids may be regulated through the phosphorylation of BES1 in plants. It indicates that BES1 does not only play important roles in stress response but also influence fruit quality in tomato. However, the molecular mechanism of this candidate gene needs further functional verification.

**Figure 5:**
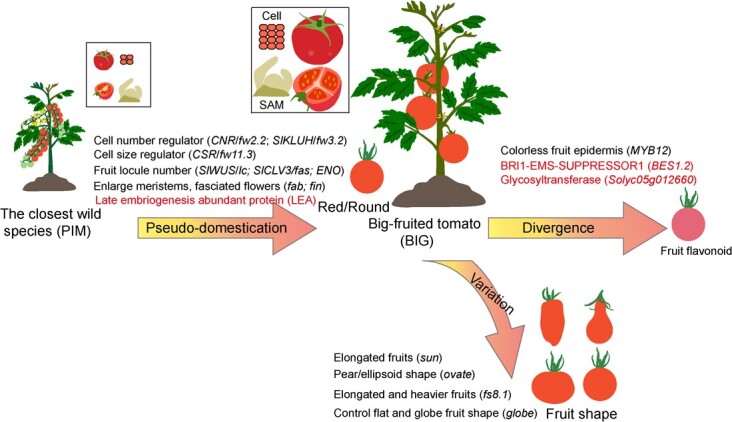
Crucial morphological and metabolic changes during tomato pseudo-domestication, divergence and variation, and the underlying key genes.

Collectively, our study indicated artificial selection for fruit mass during tomato pseudo-domestication. The divergence of flavonoid biosynthesis was clarified by the distinct differentiation among large-fruited tomatoes (Fig. 5). These findings not only provide insights into tomato pseudo-domestication and divergence, but they will also facilitate *de novo* domestication of wild relatives [43, 44] and future variome-guided tomato breeding.

## Materials and methods

### Plant sequencing and phenotyping

The resequencing data of 225 tomato accessions, including 53 *S. pimpinellifolium* (PIM, the wild accessions harboring small fruit), 166 *S. lycopersicum* (BIG, the cultivars harboring big fruit), and 6 wild accessions, were used from our previous research [2]. The important agronomic traits highly correlated to FW, including OTD, SN, SL, FSL, and FSD, were downloaded from the previous study [45]. The six kinds of flavonoid metabolites, including SIFM0967 (heptamethoxyflavone), SIFM0969 (apigenin 7-*O*-glucoside), SIFM0970 (apigenin 5-*O*-glucoside), SIFM0981 (naringenin 7-*O*-glucoside), SIFM1294 (naringenin *O*-malonyl hexoside), and SIFM1385 (tricetin *O*-malonyl hexoside), were used from our previous research [13].

### Phylogenetic analysis

A subset of 46,850 SNPs (missing data <10%, MAF >5%, and *r*^2^ < 0.2) were screened from the entire SNP data set in the 225 tomato accessions using plink software (version 1.90; https://www.cog-genomics.org/plink/1.9/) with the following parameters: —indep-pairwise 50 5 0.2 —maf 0.05 —geno 0.1. The phylogenetic tree was constructed for the accessions using phylip software (version 3.698).

### Selective sweep detection

We used three strategies to identify the selected genomic regions, including EigenGWAS, nucleotide diversity (π) analysis, and XP-CLR methods. For EigenGWAS analysis [46, 47], we used the first eigenvector of the Principal Component Analysis (PCA) as “phenotype” and performed GWAS on all tomato accessions using TASSEL software (version 5) [48] with the default parameters. The top 5% (6.398) windows were determined as candidate selected regions. For π analysis, we scanned the whole genomic regions using the PopGen module in Bioperl (version 1.7.8) [2]. The top 5% (16.466) of ratios (π_PIM_/π_BIG_) were considered as candidate selected regions. For XP-CLR analysis [49], we exploited a composite likelihood method for detecting selected sweeps between the PIM and BIG groups, with the following parameters: —maxsnps 600 —size 100 000 —step 10 000. The top 5% (21.56) of the entire genome with the highest XP-CLR values were considered as candidate regions. Finally, we merged windows that were less than 100 kb into one selected region. Genes within these selected regions were defined as pseudo-domesticated genes.

### RNA sequencing analysis

The transcriptome data of 26 PIM and 259 BIG tomato accessions from the previous study [13] were obtained to identify the DEGs using HISAT2 (version 2.1.0) [50] with the default parameters. The aligned reads are submitted to StringTie (version 2.0.3) [51] for transcript assembly with the default parameters. Finally, we filtered out genes with FPKM equal to zero in all tomato accessions and identified DEGs between the PIM and BIG groups (unpaired samples) using the DEGseq2 program. GO (gene ontology) enrichment analysis was performed using the TopGO program and KEGG enrichment analysis using the clusterProfiler program.

### Bulked segregant analysis of the F_2_ population

The data of TS-19 (PIM, 1.7 g), TS-400 (BIG, 260.1 g), and 500 F_2_ individuals were obtained from the previous study [13]. We aligned the short-read data to the reference genome using Burrows-Wheeler Aligner (version 0.7.16a) [52]. The SNPs between TS-19 and TS-400 lines were identified using samtools (version 1.5) [53] and bcftools (version 1.9) [54]. We calculated the SNP index and the mean SNP index of bulk samples using the sliding window method (window: 100 kb, step size: 10 kb).

### GWAS analysis

The SNPs were filtered with MAF >5%, missing rate <10%. After filtering, a total of 2,154,571 SNPs in the PIM and BIG groups were used for GWAS with the EMMAX software [55]. The kinship matrix was measured with the default parameter, and the first five principal components were considered as fixed effects. The suggested *P* value (2.11 × 10^−6^) and the significant *P* value (1.05 × 10^−7^) were calculated by the 474,845 effective SNPs using the GEC software (version 0.2) [56]. The significant differences of these phenotypes were measured using the ANOVA and Wilcoxon test. The correlation and Gaussian distribution of these phenotypes were analyzed and displayed using the PerformanceAnalytics program.

### Quantitative real-time PCR (qRT-PCR) analysis

We extracted the total RNA of the fruit ovary in the pre-anthesis (I), full-bloom (II), 5 days post-anthesis (III), and 10 days post-anthesis (IV) stages using the Quick RNA Isolation Kit (Huayueyang Biotechnology Company). The relative expression of target genes was calculated through the 2^-^$ ^{\triangle\triangle} $^Ct^, and the *SlEXP* (*Solyc07g025390*) gene was used as an internal control. The significant differences were calculated according to Student *t* test.

### Phylogenetic analysis of BES1 proteins

We retrieved eight BES1-related homologous genes of tomato (*S. lycopersicum*) [57], *Solanum tuberosum* [58], *A. thaliana* [29], *O. sativa* [59], *Z. mays* [60], and *Medicago polymorpha* [61]. The multiple alignment was performed using the MUSCLE algorithm with default setting [62]. We built a phylogenetic tree using the neighbor joining method from the MEGA (version X) software [63] with 1000 bootstraps.

### Dual-luciferase assay

We constructed the reporter construct (*proSlMYB12*-LUC) through transferring the promoter sequence of *SlMYB12* into the pGreenII 0800-LUC vector, and two different effector constructs through inserting the coding sequences of *SlBES1.2* into pGambia1300-GFP. The GV3101 (*Agrobacterium tumefaciens* strain) carrying the above constructs was injected into the young leaves of *N. benthamiana*. After 2 days, the LUC and REN activities in the leaves were measured using the Dual-Luciferase Reporter Assay Kit (Vazyme, DL101-01) in three separate experiments. The results were inferred from at least three biological replicates in each experiment.

### Overexpression vector construction and plant transformation

We transferred the full-length CDSs of *SlBES1.2*^167S^ and *SlBES1.2*^167P^ into pDONR221 using Gateway BP Clonase II (Invitrogen, 11 789 020). Then, their sequences were reconstituted into the destination vector pH7WG2D using Gateway LR Clonase II (Invitrogen, 11 791 020). The *A. tumefaciens* strain EHA105 carrying the final vector infected the tomato fruit (Micro Tom) at the breaker stage [64]. After 6 days, the total RNA of transgenic fruit pericarp tissues was extracted, and the expression levels of these genes were measured using qRT-PCR analysis. The primer sequences used in this study were shown in Supplemental Table 13.

## Supplementary Material

Web_Material_uhad142

## Data Availability

Raw sequence data reported in this study have been deposited in the NCBI Sequence Read Archive under the accession number SRP045767. The RNA-seq data have been deposited under an NCBI BioProject accession PRJNA396272. The complete phenotype data set is also available in ref. 15.
