# Supplementary material for: Genomic basis of selective breeding from the closest wild relative of large-fruited tomato
Source: Hortic Res. 2023 Jul 8;10(8):uhad142. doi: 10.1093/hr/uhad142 (PMC10410300; doi:10.1093/hr/uhad142)
Supplement: Web_Material_uhad142 [file web_material_uhad142.zip › Supplementary Information.docx]

**Supplementary Information**

**Supplementary Tables**

**Supplementary Table 1.** Putative selection sweeps by EigenGWAS in PIM-BIG groups.

**Supplementary Table 2.** Putative selection sweeps by nucleotide diversity (π) in PIM-BIG groups.

**Supplementary Table 3.** Putative selection sweeps by XP-CLR in PIM-BIG groups.

**Supplementary Table 4.** Putative selection sweeps by three strategies in PIM-BIG groups.

**Supplementary Table 5.** Genes within the putative EigenGWAS selection sweeps.

**Supplementary Table 6.** Genes within the putative nucleotide diversity (π) selection sweeps.

**Supplementary Table 7.** Genes within the putative XP-CLR selection sweeps.

**Supplementary Table 8.** Summary of 44 genes/QTLs related to tomato fruit weight.

**Supplementary Table 9.** GO enrichment analysis of DEGs.

**Supplementary Table 10.** KEGG enrichment analysis of DEGs.

**Supplementary Table 11.** List of lead SNPs significantly associated with six fruit weight related agronomic traits and the related candidate genes.

**Supplementary Table 12.** Six flavonoids were highly correlated with fruit weight in BIG group.

**Supplementary Table 13.** The primers of related genes used in this study.

**Supplementary Figures**

**Supplementary Figure 1. The phylogenetic tree based on the 46,850 SNPs of the 225 accessions.** The colors indicate the PIM (green), BIG (blue) and wild (purple) lines.

**Supplementary Figure 2. Heat map for differentially expressed genes (DEGs) between the PIM and BIG groups.**

**Supplementary Figure 3. Enrichment analysis of DEGs within pseudo-domestication sweeps in tomato.** The Gene ontology (GO) enrichment analysis (**A**) and KEGG pathway enrichment analysis (**B**) for DEGs within the pseudo-domestication sweeps between the PIM and BIG groups.

**Supplementary Figure 4. Sampling period and loculus of the tomato.** The ovary sampling in the for Pre-anthesis (I), Full-bloom stage (II), 5 days post anthesis (III) and 10 days post anthesis (IV) (**A**). Cross section of fruits of different tomato varieties at green and red ripening stages (**B**). Statistical analysis of the locule number among three genotypes tomato (**C**).

**Supplementary Figure 5. Statistical analysis of several agronomic traits in the PIM and BIG groups.** The significance of the differences in these traits between the PIM and BIG groups were tested by Wilcoxon test. These traits including FW, OTD, SN, SL, FSD, FSL and LN.
