# Supplementary figures and images for: Genomic basis of selective breeding from the closest wild relative of large-fruited tomato
Source: Hortic Res. 2023 Jul 8;10(8):uhad142. doi: 10.1093/hr/uhad142 (PMC10410300; doi:10.1093/hr/uhad142)

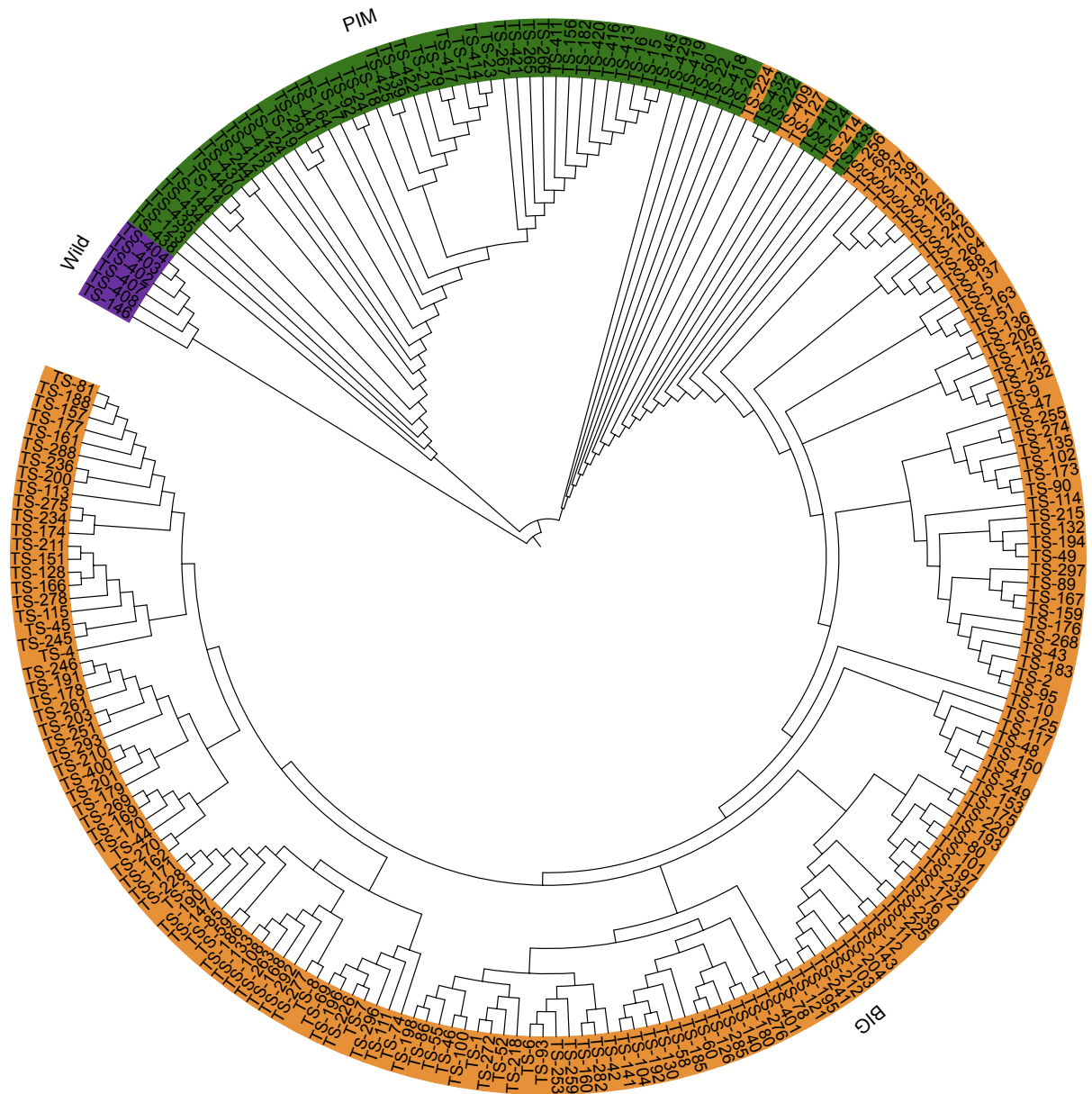

Supplement: Web_Material_uhad142 [file web_material_uhad142.zip › Supplemental Figure 1.pdf]

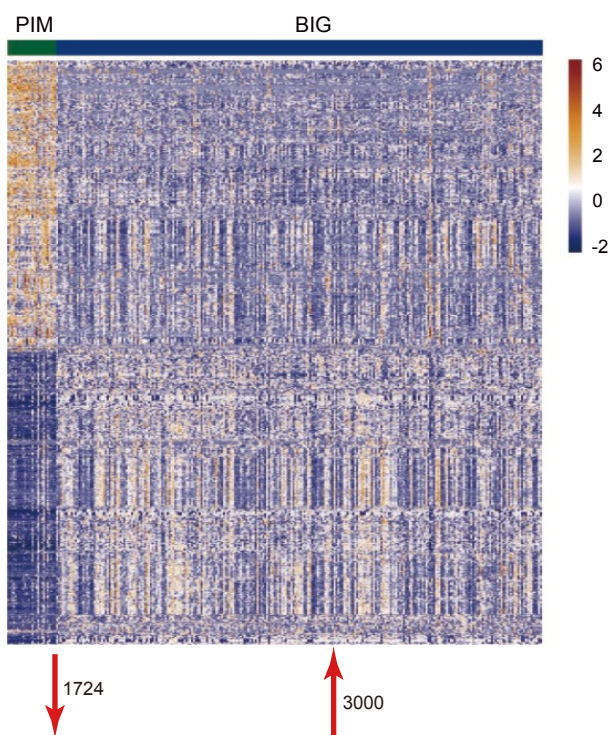

Supplement: Web_Material_uhad142 [file web_material_uhad142.zip › Supplemental Figure 2.pdf]

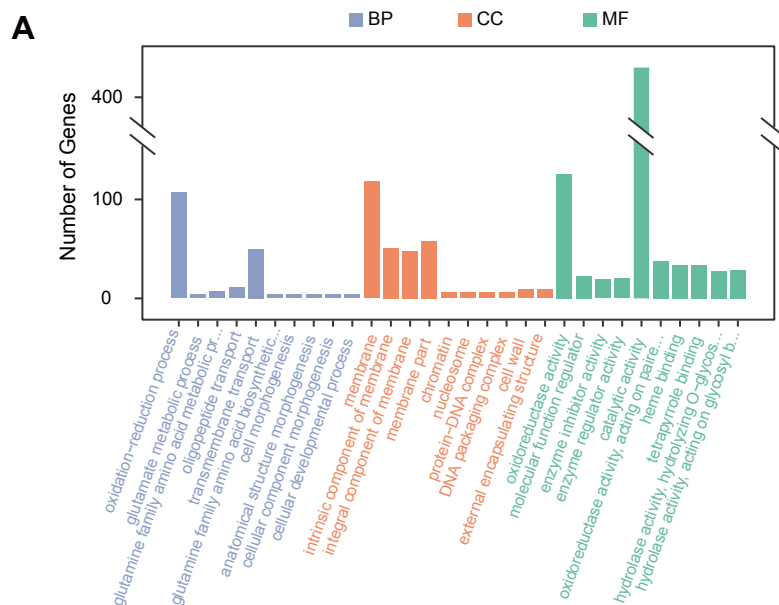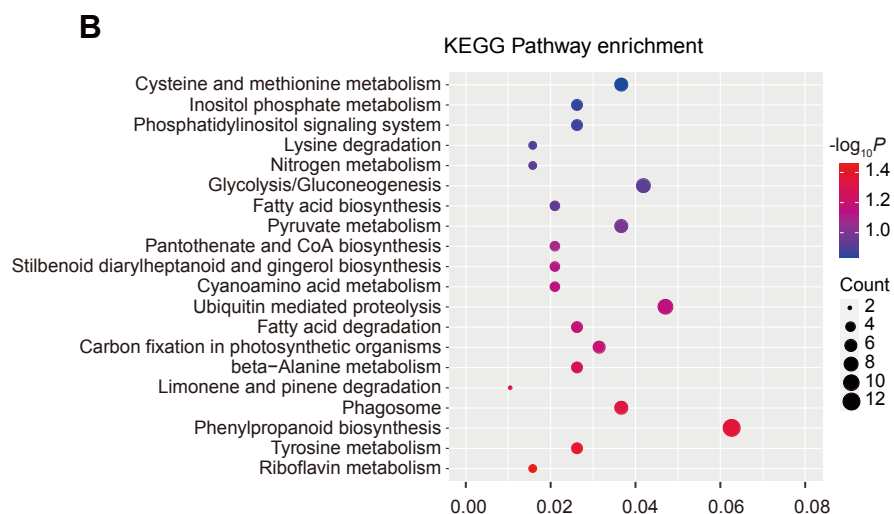

Supplement: Web_Material_uhad142 [file web_material_uhad142.zip › Supplemental Figure 3.pdf]

**A**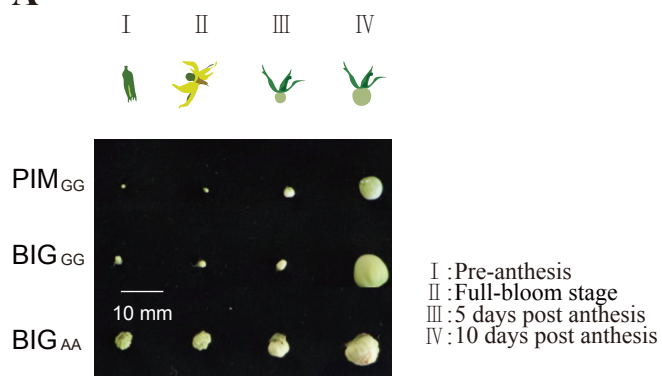**B**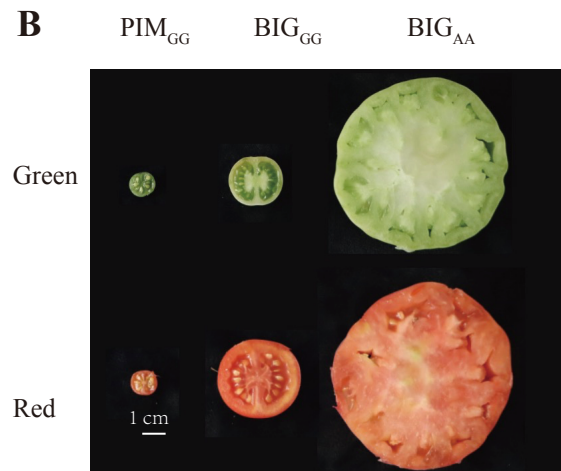**C**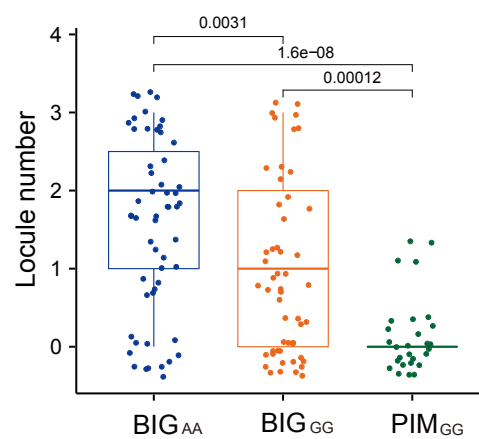

Supplement: Web_Material_uhad142 [file web_material_uhad142.zip › Supplemental Figure 4.pdf]

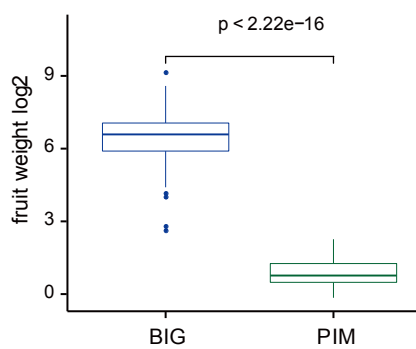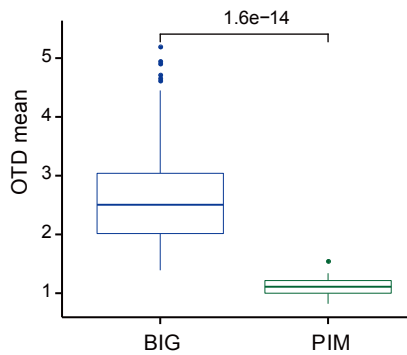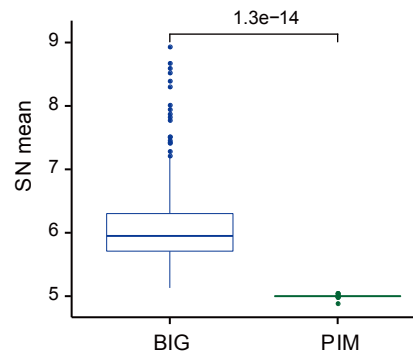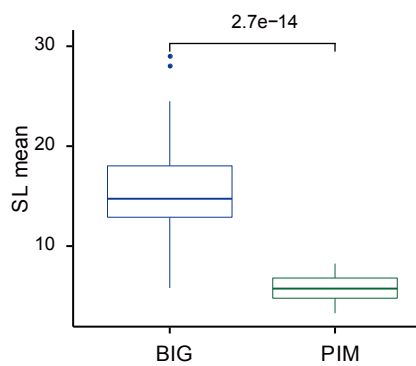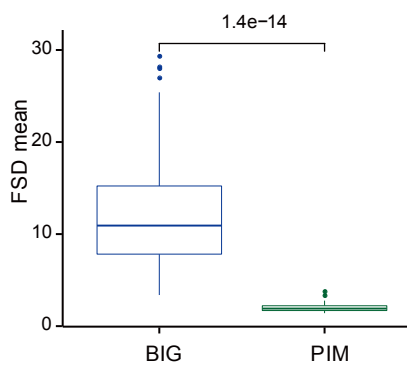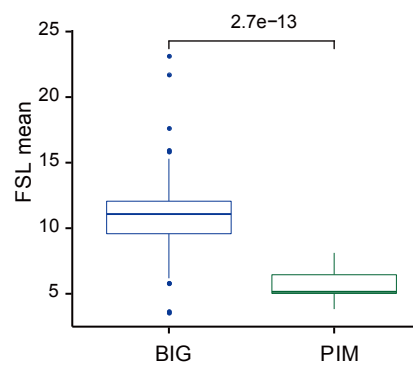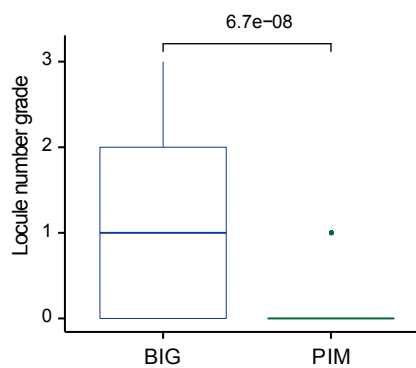

Supplement: Web_Material_uhad142 [file web_material_uhad142.zip › Supplemental Figure 5.pdf]
